# Arabidopsis Raf-Like Mitogen-Activated Protein Kinase Kinase Kinase Gene *Raf43* Is Required for Tolerance to Multiple Abiotic Stresses

**DOI:** 10.1371/journal.pone.0133975

**Published:** 2015-07-29

**Authors:** Nasar Virk, Dayong Li, Limei Tian, Lei Huang, Yongbo Hong, Xiaohui Li, Yafen Zhang, Bo Liu, Huijuan Zhang, Fengming Song

**Affiliations:** National Key Laboratory for Rice Biology, Institute of Biotechnology, Zhejiang University, Hangzhou, 310058, China; Texas Tech University, UNITED STATES

## Abstract

Mitogen-activated protein kinase (MAPK) cascades are critical signaling modules that mediate the transduction of extracellular stimuli into intracellular response. A relatively large number of MAPKKKs have been identified in a variety of plant genomes but only a few of them have been studied for their biological function. In the present study, we identified an Arabidopsis Raf-like MAPKKK gene *Raf43* and studied its function in biotic and abiotic stress response using a T-DNA insertion mutant *raf43-1* and two *Raf43*-overexpressing lines Raf43-OE#1 and Raf43-OE#13. Expression of *Raf43* was induced by multiple abiotic and biotic stresses including treatments with drought, mannitol and oxidative stress or defense signaling molecule salicylic acid and infection with necrotrophic fungal pathogen *Botrytis cinerea*. Seed germination and seedling root growth of *raf43-1* were significantly inhibited on MS medium containing mannitol, NaCl, H_2_O_2_ or methyl viologen (MV) while seed germination and seedling root growth of the Raf43-OE#1 and Raf43-OE#13 lines was similar to wild type Col-0 under the above stress conditions. Soil-grown *raf43-1* plants exhibited reduced tolerance to MV, drought and salt stress. Abscisic acid inhibited significantly seed germination and seedling root growth of the *raf43-1* line but had no effect on the two *Raf43*-overexpressing lines. Expression of stress-responsive *RD17* and *DREB2A* genes was significantly down-regulated in *raf43-1* plants. However, the *raf43-1* and *Raf43*-overexpressing plants showed similar disease phenotype to the wild type plants after infection with *B*. *cinerea* or *Pseudomonas syringae* pv. *tomato* DC3000. Our results demonstrate that *Raf43*, encoding for a Raf-like MAPKKK, is required for tolerance to multiple abiotic stresses in Arabidopsis.

## Introduction

Unlike animals, plants are sessile and cannot avoid unfavorable environmental stresses by moving to a different location; however, they have evolved a set of complicated mechanisms to timely sense and effectively respond to abiotic and biotic stresses such as drought, high salinity and extreme temperatures as well as pathogen infection and herbivore infestation [1–3]. Sensing and recognition of extracellular environmental stimuli and invading pathogens through surface/extracellular receptors such as receptor-like kinases and receptor-like proteins often activate complex downstream signaling networks, which ultimately initiate effective intracellular responses [2]. During these signaling processes, mitogen-activated protein kinase (MAPK) cascades play important roles in relaying and amplifying stimulus-specific signals to the cellular machinery through modifying a set of specific target proteins via the way of phosphorylation [4–7].

The MAPK cascade is highly conserved across different species and comprises three functional protein kinases, i.e. MAPK kinase kinases (MAPKKKs), MAPK kinases (MAPKKs) and MAPKs [4]. Compared with relatively small numbers of the MAPK and MAPKK families (e.g. 20 MAPKs and 10 MAPKKs in Arabidopsis) [8, 9], MAPKKKs form a larger family that contain more than 70 members in Arabidopsis, rice, maize, tomato, canola and *Gossypium raimondii* [10–15]. MAPKKKs act biochemically and functionally at the top of MAPK cascades but they have more variable structures and domain compositions when compared with MAPKs and MAPKKs. Based on the sequences in the kinase catalytic domain, the plant MAPKKKs are divided into three subfamilies, namely, MEKK, Raf and ZIK [10] or three groups (Group A, B and C) [8]. There are 21 MEKKs, 48 Rafs and 11 ZIKs among 80 MAPKKKs in Arabidopsis [10].

Compared with the extensive studies exploring the biological functions for the MAPKs and MAPKKs, functional characterization of MAPKKKs is relatively lagging. Recent studies using loss-of-function and gain-of-function mutants have demonstrated that MAPKKKs play important roles in growth and development as well as in abiotic and biotic stress response. In Arabidopsis, at least four MAPKKKs belonging to the MEKK subfamily have been shown to be involved in regulating growth and development, e.g. ANP1, ANP2 and ANP3 regulate cell division while YODA controls stomatal development [16–18]. The Arabidopsis MEKK1 forms MEKK1–MKK1/2–MPK4 cascade that negatively regulates defense responses against biotrophic pathogens but positively regulates defenses against necrotrophic fungi and is also involved in stress response and diverse development process [19–24]. Several MAPKKKs from other plants such as tomato MAPKKKα and MAPKKKε, tobacco NPK1, *Nicotiana benthamiana* NbMAPKKKα, NbMAPKKKβ and NbMAPKKKγ and alfalfa OMTK1 have also been implicated in regulating immune response, programmed cell death and development process [25–30]). More recently, it was found that a RXLR effector PexRD2 from *Phytophthora infestans* could target to *N*. *benthamiana* MAPKKKε to perturb plant immunity-related signaling [31].

Among the MAPKKK families characterized in plants at genome-wide level so far, approximately 60% of the members belong to the Raf subfamily; for instances, 48 Rafs out of total 80 MAPKKKs in Arabidopsis [10], 45 of 75 in rice [11], 46 of 74 in maize [12] and 39 of 66 in canola [14]. However, only a small fraction of the Raf-like MAPKKKs have been identified and studied at molecular and genetic levels for their biological functions. The biological importance of the Raf-like MAPKKKs was highlighted by the molecular identification and detailed functional analyses of Constitutive Triple Response 1 (CTR1) and ENHANCED DISEASE RESISTENCE1 (EDR1) in Arabidopsis [32, 33]. CTR1 is a Raf-like MAPKKK that modulates a negative regulation of ethylene pathway by acting upstream of MKK9-MPK3/6 proteins [34–36] and is also involved in sugar response [37]. EDR1, another Raf-like MAPKKK, functions at the top of a MAPK cascade to regulate negatively the salicylic acid (SA)-inducible defense responses [33] and directly associates with MKK4/MKK5 for negatively regulating innate immunity [38]. The Arabidopsis SIS8/AT6 was recently reported to play roles in salt stress and sugar responses [39, 40], whereas MAP3Kδ4 was shown to regulate growth and shoot branching [41]. In rice, a Raf-like MAPKKK DSM1 is involved in drought tolerance [42] while another Raf-like MAPKKK ILA1 regulates mechanical tissue formation in the leaf lamina joint [43]. The rice EDR1 was found to negatively regulate rice bacterial resistance via activation of ethylene biosynthesis [44]. However, the functions of most other Raf-like MAPKKKs in plants are as yet unknown.

In bioinformatics analysis of publicly available microarray data to identify putative stress-responsive MAPKKKs in Arabidopsis, we found that a gene, *Raf43*, encoding for a Raf-like MAPKKK, could be induced by drought, salt and oxidative stress, pathogen infection and elicitor treatment. In the present study, we performed functional analyses using a T-DNA insertion mutant line *raf43-1* and two *Raf43*-overexpresing lines Raf43-OE#1 and Raf43-OE#13 to explore the possible roles of *Raf43* in abiotic and biotic stress response. Our results showed that *Raf43* could be induced by multiple abiotic and biotic stresses and that the *raf43-1* mutant plants exhibited reduced tolerance to osmotic, drought, salt and oxidative stresses while the *Raf43*-overexpressing plants did not show any altered stress response. The *raf43-1* mutant showed increased sensitivity to abscisic acid (ABA) and the *raf43-1* plants constitutively down-regulated the expression of stress-responsive gene. However, the *raf43-1* mutant and the *Raf43*-overexpressing plants showed similar disease phenotype to the wild type plants in response to infection with necrotrophic fungal pathogen *B*. *cinerea* or bacterial pathogen *Pseudomonas syringae* pv. *tomato* DC3000. These findings demonstrate that *Raf43*, encoding for a Raf-like MAPKKK, is required for tolerance to multiple abiotic stresses in Arabidopsis.

## Material and Methods

### Plant growth and treatments

*Arabidopsis* plants were grown in a mixture of perlite: vermiculite: plant ash with a ratio of 1:6:2, respectively, under fluorescent light (200 μE·m^2^·s^-1^) at 22°C with 60% relative humidity. Seed dormancy was broken by soaking the seeds in water for 2 days in a 4°C refrigerator and then used for experiments. Four-week-old plants (ecotype Col-0) were used for different treatments to analyze the expression of *Raf43* in response to stresses. For drought treatment, rosette leaves were cut and placed on laboratory bench under similar conditions as control intact plants. For mannitol treatment, plants were irrigated with 350 mM mannitol or water as a control. For treatment with methyl viologen (MV) and salicylic acid (SA), solutions of 50 μM MV, 1 mM SA or same volume of water as a control were sprayed onto the plants. Inoculation of *B*. *cinerea* was conducted as below. Leaf samples were taken at indicated time points after treatment/inoculation and stored at -80°C until use.

### Characterization of *raf43-1* mutant

A T-DNA insertion line FLAG_505A06 for *Raf43* in Ws-0 background was obtained from Arabidopsis thaliana Resource Centre for Genomics at the Versailles Genetics and Plant Breeding Laboratory, France. A three-primer PCR-based genotyping strategy was conducted using gene-specific primers FLAG_505A06-LP (GCG CAG TTG ATT ATA CGA GAA G) and FLAG_505A06-RP (GCG AGG AGG AGT ACT GTG ATG) with a T-DNA primer F-LB4 (CGT GTG CCA GGT GCC CAC GGA ATA GT) to identify wild type, heterozygous and homozygous individuals. Plants homozygous for the *Raf43* mutation were used for further experiments and this line was designated as *raf43-1*.

### Generation of *Raf43*-overexpressing and lines

For construction of overexpression vector, a 1450 bp fragment of *raf43* gene was amplified using a pair of primers Raf43-1F (ATG GAT GGA GAG GTT ACT TCT TGG A) and Raf43-1R (CAC AAA AAT GAG TGA CTC TAG ACG T) and cloned into pUC19 by T/A cloning, yielding a recombinant plasmid pUC19-Raf43. After confirmation by sequencing, the open reading frame (ORF) of *Raf43* was amplified using a pair of primers Raf43-2F (AGT GGA TCC ATG GAT GGA GAG GTT ACT TCT, a *Bam*HI site underlined) and Raf43-2R (GCA GTC GAC TCA AGC GAA TTT AGG CTT AGGT, a *Sal*I site underlined) with the recombinant plasmid pUC19-Raf43 as template and cloned into binary vector pCAMBIA 99–1, resulting a recombinant plasmid pCAMBIA991-Raf43. For construction of complementation vector, the *Raf34* ORF was amplified from pUC19-Raf43 using primers Raf43-2F and Raf43-2R and cloned into pCAMBIA 1301 at *Bam*HI/*Sal*I sites, yielding plasmid pCAMBIA1301-Raf43. A 1295 bp fragment upstream the start codon of the *Raf43* gene was amplified from genomic DNA using a pair of primers pRaf43-1F (AGT GAATTC AGCAGGGAGAGGGATAGGGT, a *Eco*RI site underlined) and pRaf43-1R (AGT GGATCC GTTTAAACCCCTCCCCAAAAC, a *Bam*HI site underlined) and cloned into pCAMBIA1301-Raf43 at *Eco*RI/*Bam*HI sites, yielding plasmid pCAMBIA1301-proRaf43-Raf43. Plasmids pCAMBIA991-Raf43 and pCAMBIA1301-proRaf43-Raf43 were introduced into *Agrobacterium tumefaciens* strain GV3101 by electroporation using GENE PULSER II Electroporation System (Bio-Rad Laboratories, Hercules, CA, USA). Arabidopsis transformation was performed using the floral dip method [45]. The overexpression plasmid pCAMBIA991-Raf43 was introduced into Col-0 background while the complementation plasmid pCAMBIA1301-proRaf43-Raf43 was introduced into *raf43* background (harboring Basta resistance selection marker). Seeds from transformed plants (T0) were harvested and sown on 1/2 MS supplemented with 50 μg/mL hygromycin (Hgr). Seedlings of the T1 generation were selected and self-pollinated. T1 progenies on selective medium with Hgr-resistant/Hgr-sensitive segregating ratio of 3:1 were selected and transformed to soil for self-pollination. Progenies of the individual T1 plants were observed on selective medium and those showing 100% resistance to Hgr were selected as homozygous lines. Homozygous overexpression lines Raf43-OE#1 and Raf43-OE#13 and homozygous complementation lines Raf43-C1 and Raf43-C17 were used for further studies.

### Abiotic stress assays

In seed germination and root growth assays, seeds were surface-sterilized and plated on 1/2 MS medium supplemented with or without different concentrations of mannitol, NaCl, MV, H_2_O_2_, or ABA and incubated at 4°C for 2 days to synchronize germination. The plates were placed in a growth chamber under long day condition and germination was counted at 7 days after incubation. Root growth of the seedlings was measured after placing the Petri dishes vertically for 10 days. In whole plant abiotic stress assays, three-week-old soil-grown plants were used. Drought stress was applied to plants by withholding water for 7–10 days while salt stress was applied to plants by drenching with solution of 200 mM NaCl. Oxidative stress treatment was conducted by foliar spraying with 50 mM MV onto leaves of the plants. After a period of treatment, the numbers of plants that continued to grow were recorded and the survival rate was calculated. In these abiotic stress assays, plants grown under normal condition or without abiotic stress treatment were used as controls. Chlorophyll content of leaf tissues of the plants in salt and oxidative stress assays were measured according to the method described previously [46]. Chlorophyll content was calculated according to the formula Chl (A+B) = 5.24A_664_+22.24A_648_, where Chl is the chlorophyll concentration in micrograms per milliliter and A is the absorption. Relative water content (RWC) in leaves of the plants in drought stress assays was measured according to the method described previously [47]. Fully expanded leaves were detached from 6 individual plants to measure the leaf fresh weight (W_F_), turgid leaf weight (W_T_), and dry weights (W_D_) and RWC were calculated from the equation RWC (%) = (W_F_-W_D_)/(W_T_-W_D_)×100% [47].

### Disease Assays

Disease assays with *B*. *cinerea* were performed according to previously reported procedure [46]. *B*. *cinerea* was grown on 2 × V8 agar (36% V_8_ juice, 0.2% CaCO_3_ and 2% agar) at 22°C for 10–12 days. Spores were collected by dissolving in 1% maltose media and filtered through bi-layered cheesecloth. Spore density in spore suspension was adjusted to 1×10^5^ spores/mL. In detached leaf inoculation assays, fully expanded leaves from at least 10 individual plants were inoculated by dropping a 5 μL of spore suspension onto leaf surface. In whole plant inoculation assays, 4-week-old plants were inoculated by foliar spraying with spore suspension or buffer (as a mock-inoculation control). The inoculated leaves and plants were kept in sealed trays or tanks with high relative humidity at 22°C to facilitate disease development. The diameter of each lesion in detached leaf inoculation assays was measured at 5 days after inoculation.

*P*. *syringae* was cultivated on plates of King’s B (KB) medium supplemented with 50 mg/mL rifampicin and resuspended into 10 mM MgCl_2_ at OD_600_ = 0.002 for plant inoculation. Four-week-old plants were infiltrated at two sites of leaves with 20 μL aliquots of the bacterial suspension using 1-ml syringes without needles. After inoculation, plants were covered with a transparent plastic film and disease symptoms were observed daily. For measurement of bacterial titers in inoculated leaves, leaf punches (6 mm in diameter) were collected at 0, 2 or 4 days post infiltration, surface sterilized in 70% ethanol for 10 seconds, homogenized in 200 μL of 10 mM MgCl_2_, diluted in 10 mM MgCl_2_, and plated on KB agar plates containing rifampicin at 100 μg/mL.

### Analyses of gene expression

For Genevestigator-based analyses, microarray expression data from various datasets were obtained using GENEVESTIGATOR (https://www.genevestigator.com/gv/). The expression data for *Raf43* (Locus At3g46930) under different abiotic and biotic stress conditions were mined. Most of the expression data were mined from ecotype Col-0 except for a set of the expression data in *P*. *syringae* pv. *tomato* DC3000 experiments was obtained from ecotype Ws-0. Basically, only expression changes of *Raf43* in stress/elicitor-treated or pathogen-infected plants >2 folds over controls with *p* values <0.05 were used. The microarray expression analyses were obtained from samples treated with cold, drought, osmotic, oxidative or salt stresses, infected by pathogens such as *Alternaria brassicicola* (*Ab*), *B*. *cinerea* (*B*c), *Blumeria graminis* f. sp. *hordei* (*Bg*), *P*. *syringae* pv. *maculicola* (*Psm*), *P*. *syringae* pv. *phaseolicola* (*Psp*), *P*. *syringae* pv. *tomato* (*Pst*) DC3000, or *Sclerotinia sclerotiorum* (*Ss*) or treated with elicitor including fungal chitin, bacterial EF-Tu (elf18 and elf26), FLG22 and hrpZ, Arabidopsis Pep2, and chemical inducer SA. For qRT-PCR-based analyses, total RNA was extracted from leaf samples using TRIZOL reagent (Invitrogen, Shanghai, China) and treated using DNase RQ1 (Promega, Madison, USA) to remove contaminant genomic DNA. First-strand cDNA was reversely transcribed using AMV reverse transcriptase (Takara, Dalian, China) with oligod(T) primer according to the manufacturer’s instructions. Primers used in qRT-PCR-based analyses of gene expression were Raf43-3F, TCG TCG TCC CGC AAA GAT; Raf43-3R, TTC CCG TGA GCA AAC CTA; AtABA2-F, CTC GCT TTG GCT CAT TTG C; AtABA2-R, CCG TCA GTT CCA CCC CTT T; AtNCED3-F, CCG GTG GTT TAC GAC AAG AA; AtNCED3-R, CCC AAG CGT TCC AGA GAT G; AtRD17-1F, ACG TCC ACG CCG TTG GT; AtRD17-1R, CTC CGG ATG TTC CAC TGG AA; AtDREB2A-1F, GAC CTA AAT GGC GAC GAT GT, AtDREB2A-1R, TCG AGC TGA AAC GGA GGT AT; AtUBC-1F: TCA AAT GGA CCG CTC TTA TC; AtUBC-1R, CAC AGA CTG AAG CGT CCA AG. qRT-PCR was conducted with SYBR premix Ex Taq (Takara, China) in a CFX96 Real-Time System (Bio-Rad, Hercules, CA, USA) according to the manufacturer’s instructions. The expression levels were quantified using 2^−ΔΔCT^ method. The data obtained from qRT-PCR were normalized using the levels of the *UBC* gene and the gene expression levels in *raf43-1*, *Raf43*-overexpressing or treated/inoculated plants were shown as folds of the levels in the control untreated or mock-inoculated plants, which were set as 1.0.

### Statistical analysis

All experiments were repeated independently for three times and at least 10 plants were included in each treatment in an independent experiment. Data obtained from three biological replications were subjected to statistical analysis by the Student’s *t*-test method using DPS software (http://www.chinadps.net/index.htm) and the results are presented as mean ± standard errors. The probability values of *p*<0.05 were considered as significant between different treatments.

## Results

### *Raf43* is responsive to multiple abiotic and biotic stresses

**Fig 1:**
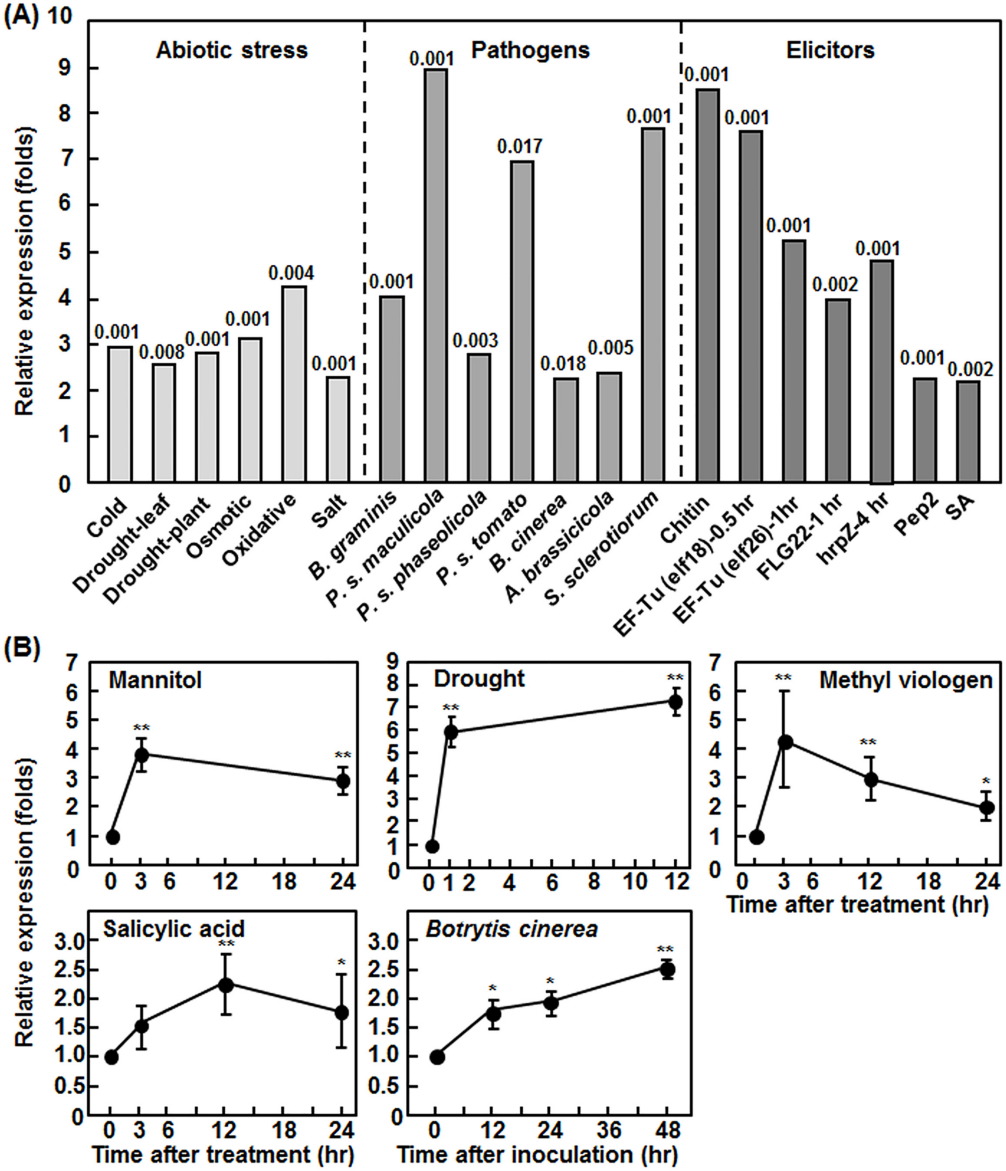
Responsiveness of *Raf43* to biotic and abiotic stresses. (A) Genevestigator-based analysis of expression profile of *Raf43* in response to abiotic stress, pathogen infection and elicitor treatment. Expression data were extracted from Genevestigator microarray datasets obtained from experiments with ecotype Col-0 plants except for the *P*. *syringae* pv. *tomato* experiment, which was conducted with Ws-0 plants. The *p* values for the expression data between the treated/inoculated and control plants are shown above the columns. *B*. *graminis*, *Blumeria graminis*; *P*. *s*. *maculicola*, *Pseudomonas syringae* pv. *maculicola*; *P*. *s*. *phaseolicola*, *P*. *syringae* pv. *phaseolicola*; *P*. *s*. *tomato*, *P*. *syringae* pv. *tomato* DC3000; *B*. *cinerea*, *Botrytis cinerea*; *A*. *brasicicola*, *Alternaria brassicicola*; *S*. *sclerotiorum*, *Sclerotinia sclerotiorum*. (B) qRT-PCR-based analysis of expression patterns of *Raf43* in response to treatments with mannitol, drought, methyl viologen or salicylic acid and to infection by *B*. *cinerea*. The expression level of *Ubiquitin* was used to normalize the *Raf43* expression data and the relative expression was calculated as folds of expression in treated/inoculated plants vs. those in the control plants. Experiments were repeated with three independent biological samples at each time point and data represented are the means ± standard errors from three independent experiments. * and ** indicate the significant difference at *p* = 0.05 and *p* = 0.01, respectively, as compared with those in the controls.

In our bioinformatics analyses of expression profiles of putative stress-responsive MAPKKKs, we found that a Raf-like MAPK kinase kinase gene, designated as *Raf43*, was highly induced by abiotic and biotic stresses. To analyze in detail the expression profile of *Raf43* under stress conditions, we extracted the expression data of *Raf43* (At3g46930) from public microarray databases using GENEVESTIGATOR platfrom. In our analyses, only expression changes of *Raf43* in stress/elicitor-treated or pathogen-infected wild type Col-0 plants (except the *Pst* DC3000 experiment which used wild type Ws-0 plants) >2 folds with *p* values <0.05 were used to generate the expression profile of *Raf43* in response to abiotic stress, pathogen infection and elicitor treatments (Fig 1A). Under abiotic stress conditions such as cold, drought, oxidative, osmotic and salt treatments, the expression levels of *Raf43* in Col-0 plants were significantly increased by 2.26~4.23 folds over those in the controls (Fig 1A). In response to infection by different types of pathogens, e.g. biotrophic/hemibiotrophic (e.g. *B*. *graminis* and *P*. *syringae*) vs. necrotrophic (e.g. *B*. *cinerea*, *A*. *brassicicola* and *S*. *sclerotiorum*) and fungal (e.g. *B*. *graminis*, *B*. *cinerea*, *A*. *brassicicola* and *S*. *sclerotiorum*) vs. bacterial (e. g. *P*. *syringae* pvs. *maculicola*, *phaseolicola* and *tomato*) pathogens, the expression of *Raf43* was significanlty induced in Col-0 and Ws-0 (only for *P*. *syringae* pv. *tomato* DC3000) plants, leading to 2.26~8.91 folds of increase over those in mock-inoculated plants (Fig 1A). In particular, relatively high levels of induction of *Raf43* expression was observed after infections by bacterial pathogens *P*. *syringae* pv. *maculicola* and *P*. *syringae* pv. *tomato* DC3000 and necrotrophic fungal pathogen *S*. *sclerotiorum*, showing 8.91, 6.95 and 7.64 folds of increase, respectively (Fig 1A). Similarly, several well-known pathogen-associated molecular patterns such as fungal chitin and bacterial EF-Tu (elf18 and elf26) and FLG22, bacterial effectors such as HrpZ, Arabidopsis Pep2 protein and well-known defense signaling hormone SA, which are capable of activating both innate and inducible immune responses in Arabidopsis, induced significantly the expression of *Raf43* in Col-0 plants, giving 2.16~8.48 folds of increase over those in the untreated plants (Fig 1A). Notably, the induced expression of *Raf43* occurred rapidly in response to elicitor treatment; for example, significant induction of *Raf43* expression was detected within one hour after treatment with EF-Tu and FLG22 (Fig 1A). To further confirm the expression profile of *Raf4*3 in response to stress, we analyzed by qRT-PCR the expression patterns of *Raf43* in Col-0 plants after treatment with mannitol, drought, MV and SA and infection with *B*. *cinerea*. The expression levels of *Raf43* in mannitol- and MV-treated plants peaked with approximately 3 folds of increase over those in untreated plants at 3 hr and then decreased, whereas the expression level of *Raf43* in drought stress-treated plants markedly increased with 6 folds over that in untreated control plants at 1 hr and continuously increased during a period of 12 hr (Fig 1B). When treated with 1 mM SA, the expression level of *Raf43* increased at 3 hr, peaked with 2.35 folds of increase over that in untreated plants at 12 hr and then decreased (Fig 1B). Upon infection by *B*. *cinerea*, the expression level of *Raf43* continuously increased during a period of 48 hr after inoculation (Fig 1B). These results confirmed the inducible expression feature of *Raf43* revealed from analyses of public microarray data. It is worthy to note that the expression levels of *Raf43* induced by SA treatment and by infection of *B*. *cinerea* in the qRT-PCR experiments were comparable to the levels observed from analyses of the public microarray data (Fig 1A and 1B). Taken together, these data indicate that *Raf43* is responsive to multiple abiotic and biotic stresses and this stress-inducbile expression feature imply the involvement of *Raf43* in abiotic and biotic stress response.

### Characterization of *raf43-1* mutant and *Raf43*-overexpressing transgenic lines

**Fig 2:**
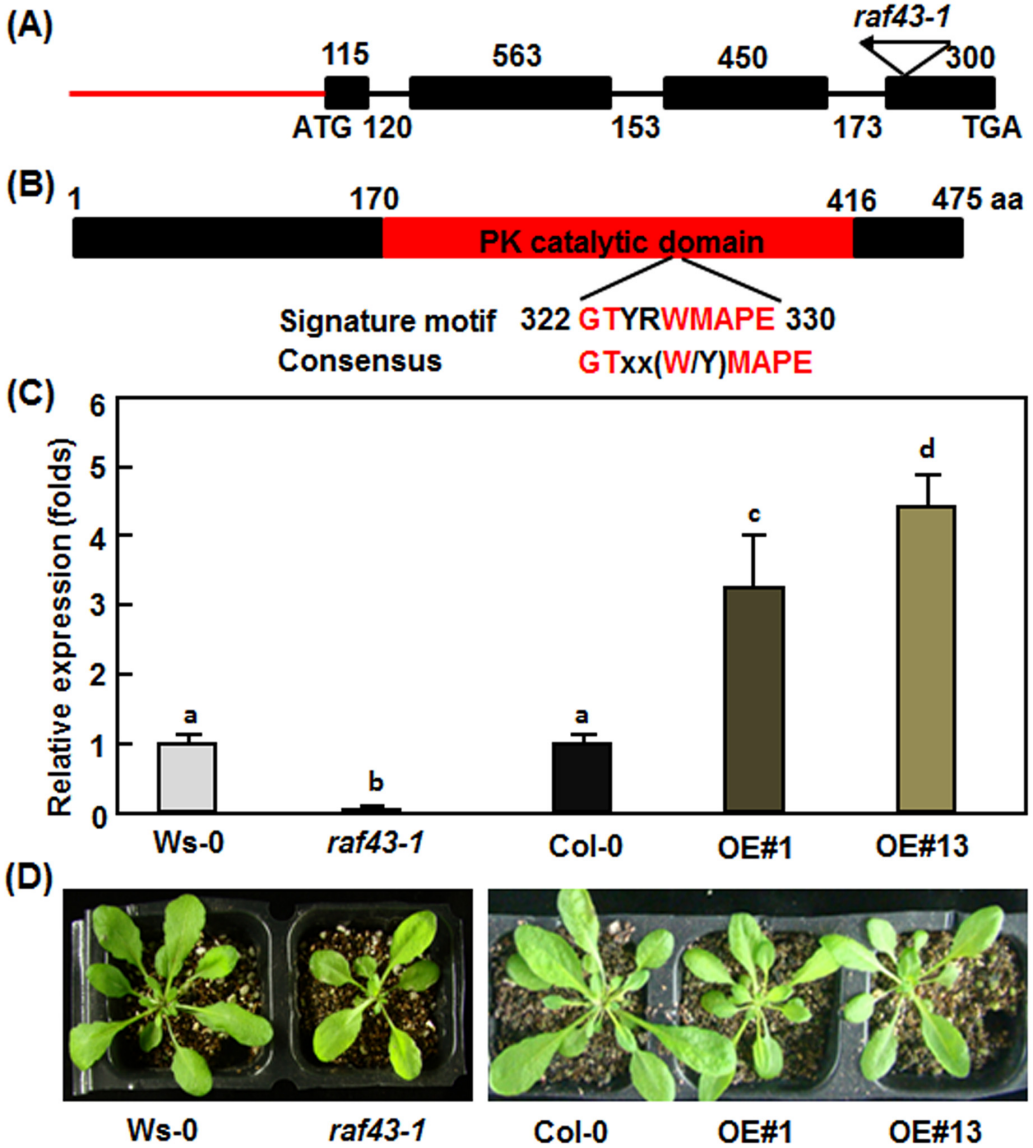
Structures of the *Raf43* gene/protein and characterization of the *raf43-1* mutant and the *Raf43*-overexpressing lines. (A) Diagram showing the structure of *Raf43* and the T-DNA location in *raf43-1* mutant. Black boxes, black lines and red line indicate the exons, introns and putative promoter region, respectively. Sizes (bp) of the exons and introns are indicated above and below the diagram, respectively. The location and orientation of the T-DNA inserted in *raf43-1* mutant line are shown. The start (ATG) and stop (TGA) codons are also indicated. (B) Diagram showing the structure of the Raf43 protein. The red box indicates the conserved protein kinase (PK) catalytic domain in Raf43 protein. Signature motif and its position in Raf43 protein and consensus of the signature motif commonly present in Raf-like MAPKKKs are shown. The amino acid positions are also indicated above the diagram. (C) Levels of the *Raf43* transcripts in *raf43-1* mutant and *Raf43*-overxpressing lines Raf43-OE#1 and Raf43-OE#13 plants. The transcript levels of *Raf43* in four-week-old plants grown under normal conditions were analyzed by qRT-PCR using *Raf43*-specific primers and data were normalized by the transcript level of *Ubiquitin*. The transcript levels of *Raf43* in wild type (Ws-0 and Col-0) plants were set as 1 and the levels in *raf43* mutant and *Raf43*-overxpressing are shown as folds of the levels in wild type plants. Data represented are the means ± standard errors from three independent experiments. Different letters above the columns indicate significant difference at *p* = 0.05 between Ws-0 and *raf43-1* plants and between Col-0 and *Raf43*-overexpressing plants. (D) Morphological and growth phenotypes of four-week-old plants of wild type (Ws-0 and Col-0), *raf43-1* mutant and *Raf43*-overexpressing lines OE#1 and OE#13.

*Raf43* is encoded by At3g46930 and the predicted ORF of *Raf43* contains 4 exons and 3 introns (Fig 2A). Our cloning and sequencing confirmed the sequence of the predicted ORF of *Raf43*. The *Raf43* gene encodes a 475 aa protein, which contains a typical protein kinase catalytic domain (170–416 aa) and a signature motif (322–330 aa) commonly present in Raf-like MAPKKKs [10] (Fig 2B). The protein structure feature indicates that Raf43 is a Raf-like MAPKKK and belongs to Group C of plants MAPKKKs [8]. To assess the biological function of *Raf43*, one T-DNA insertion line FLAG_505A06 in ecotype Ws-0 background [48] was analyzed. The T-DNA was inversely inserted in the last exon of the *Raf43* gene (Fig 2A). PCR-based genotyping using a pair of gene-specific primers and a T-DNA primer identified individual plants that were homozygous at the T-DNA insertion site in the FLAG_505A06 line and this homozygous T-DNA insertion line was designated as *raf43-1*. qRT-PCR analysis revealed that the transcript level of *Raf43* in *raf43-1* plants was significantly reduced as compared with its wild type Ws-0 plants (Fig 2C), indicating that *raf43-1* is a null mutant for *Raf43*. Meanwhile, we cloned the *Raf43* ORF and inserted into pCAMBIA-991 vector under the control of CaMV 35S promoter to make a *Raf43*-overexpressing (OE) construct. The Raf43-OE construct was introduced into wild type Col-0 plants through floral dip transformation procedure and *Raf43*-overexpressing transgenic lines were obtained. Homozygous T3 lines with a single copy of the transgene were screened based on a 3:1 Hgr-resistant/Hgr-sensitive segregation ratio on selective medium and two independent lines, named *Raf43*-overexpressing OE#1 and *Raf43*-overexpressing OE#13 (thereafter referred as to Raf43-OE#1 and Raf43-OE#13), were chosen for further studies. qRT-PCR analysis showed that the transcript levels of *Raf43* in Raf43-OE#1 and Raf43-OE#13 plants were significantly increased, leading to 3–4 folds of increase as compared with wild type Col-0 (Fig 2C). During our experiments, no any defect in growth and development was observed for the *raf43-1* mutant plants and for the Raf43-OE#1 and Raf43-OE#13 plants when compared with their corresponding wild type Ws-0 and Col-0 plants, respectively (Fig 2D).

### *Raf43* is required for osmotic stress tolerance

**Fig 3:**
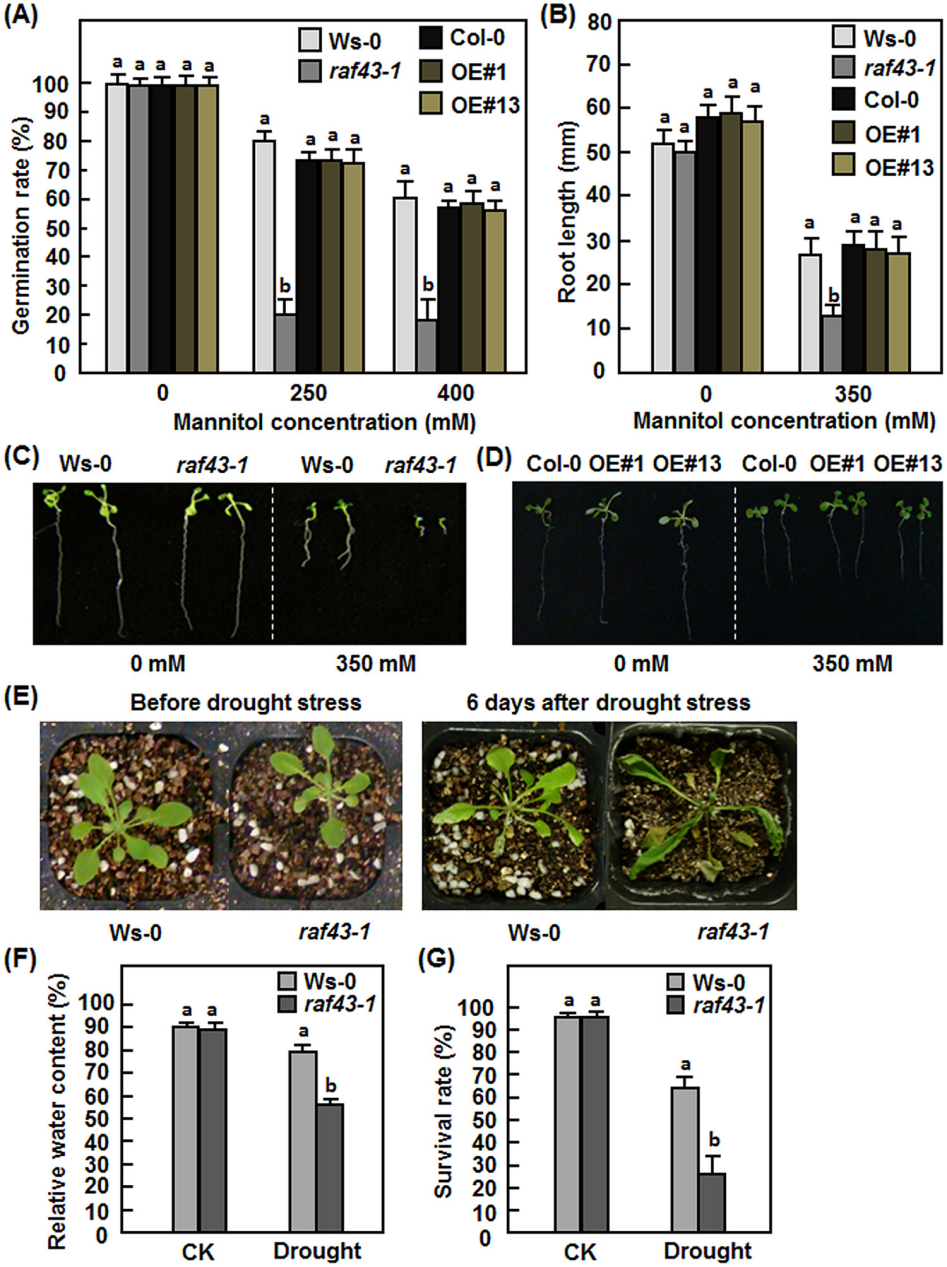
*Raf43* is required for osmotic and drought stress tolerance. (A)–(D) Seed germination and root growth of seedlings grown on 1/2 MS supplemented with different concentrations of mannitol. (A) Seed germination rates on 1/2 MS supplemented with 0, 250 and 400 mM of mannitol. Seed germination was recorded when the radicles emerged completely from seed coat. (B) Root length of seedlings grown on 1/2 MS supplemented with 0 and 350 mM of mannitol. (C) and (D) Growth phenotype of seedlings grown on 1/2 MS supplemented with 0 and 350 mM mannitol. (E)-(G) Phenotype, relative water content and survival rates of soil-grown plants after drought stress treatment. (E) Phenotype of the wild type Ws-0 and mutant *raf43-1* plants under drought stress condition. Three-week-old soil-grown plants were withheld from watering for drought stress treatment and allowed to grow for two weeks. Left photo shows the growth status of the plants before drought stress treatment while right photo shows phenotype of the plants at 7 days after drought stress treatment. (F) Relative water contents in leaf tissues of wild type Ws-0 and mutant *raf43-1* plants grown under normal and drought stress condition at 7 days after treatment. (G) Survival rates of wild type Ws-0 and mutant *raf43-1* plants grown under normal and drought stress condition at 14 days after treatment. Experiments were repeated three times and data presented in (A), (B), (F) and (G) are the means ± standard errors from three independent experiments. Different letters above the columns indicate significant difference at *p* = 0.05 between Ws-0 and *raf43-1* plants and between Col-0 and *Raf43*-overexpressing plants.

To explore the possible role of *Raf43* in abiotic stress responses, we first examined and compared the seed germination and seedling root growth of the *raf43-1* mutant line and the Raf43-OE#1 and Raf43-OE#13 lines with their corresponding wild types Ws-0 and Col-0 under osmotic stress condition mimicked by mannitol treatment. Germination rates of seeds from the *raf43-1* mutant line, the Raf43-OE#1 and Raf43-OE#13 lines and their corresponding wild type Ws-0 and Col-0 was similar on mannitol-free 1/2MS medium but the germination rates of the *raf43-1*, Raf43-OE#1, Raf43-OE#13 and wild type seeds significantly decreased on 1/2MS medium supplemented with 200 mM or 400 mM mannitol as compared with those on mannitol-free medium (Fig 3A). In contrast, germination rates of the *raf43-1* mutant seeds markedly decreased on 1/2MS medium supplemented with 200 mM or 400 mM mannitol, resulting in 70–75% of reduction as compared with the Ws-0 seeds (Fig 3A). No significant difference in germination rates of seeds from the Raf43-OE#1 and Raf43-OE#13 lines and wild type Col-0 was observed (Fig 3A). Similarly, after germination, root length of seedlings from the *raf43-1* mutant line, the Raf43-OE#1 and Raf43-OE#13 lines and their corresponding wild types Ws-0 and Col-0 was similar when grown on mannitol-free 1/2MS medium but the root growth of the *raf43-1*, Raf43-OE#1, Raf43-OE#13 and wild type seedlings was significantly inhibited when grown on 1/2MS medium supplemented with 350 mM mannitol as compared with those on mannitol-free medium (Fig 3B–3D). Root growth of the *raf43-1* seedlings was significantly inhibited on 1/2MS medium supplemented with 350 mM mannitol, leading to a 55% reduction in root length as compared with the Ws-0 seedlings (Fig 3B and 3C). No significant difference in root length of the Raf43-OE#1 and Raf43-OE#13 seedlings and the Col-0 seedlings was observed (Fig 3B and 3D). These observations indicate that knockout of *Raf43* attenuated the osmotic stress tolerance but overexpression of *Raf43* did not affect the osmotic stress tolerance, demonstrating the requirement of *Raf43* in tolerance to osmotic stress in Arabidopsis.

### *Raf43* is required for drought stress tolerance

We next examined and compared the phenotype of soil-grown three-week-old *raf43-1* mutant and its wild type Ws-0 plants in response to drought stress induced by withholding water. Before drought stress treatment, the growth status of the *raf43-1* and wild type Ws-0 plants was similar (Fig 3E, left); however, most of the leaves of the *raf43-1* plants were rolled, whereas only some old leaves of the wild type Ws-0 plants were slightly rolled at 7 days after withholding water (Fig 3E, right). Similar RWC in leaves of normally watered *raf43-1* and Ws-0 plants was observed but withholding water for 7 days significantly reduced RWC in leaves of both the *raf43-1* and Ws-0 plants (Fig 3F). However, RWC in leaves of the *raf43-1* plants (56%) was significantly lower than that in the wild type Ws-0 plants (78%), leading to a reduction of 28% in RWC (Fig 3F). After 14-day withholding water, the survival rate of the *raf43-1* plants (24%) was markedly decreased as compared with that of the wild type Ws-0 plants (63%), showing a reduction of 62% (Fig 3G). The normally watered *raf43-1* and Ws-0 plants grown in the same chamber grew well with >95% of survival rate during the 14-day experimental period (Fig 3G). These data indicate that knockout of *Raf43* resulted in reduced tolerance to drought stress, demonstrating that *Raf43* is required for drought stress tolerance in Arabidopsis.

### *Raf43* is required for salt stress tolerance

**Fig 4:**
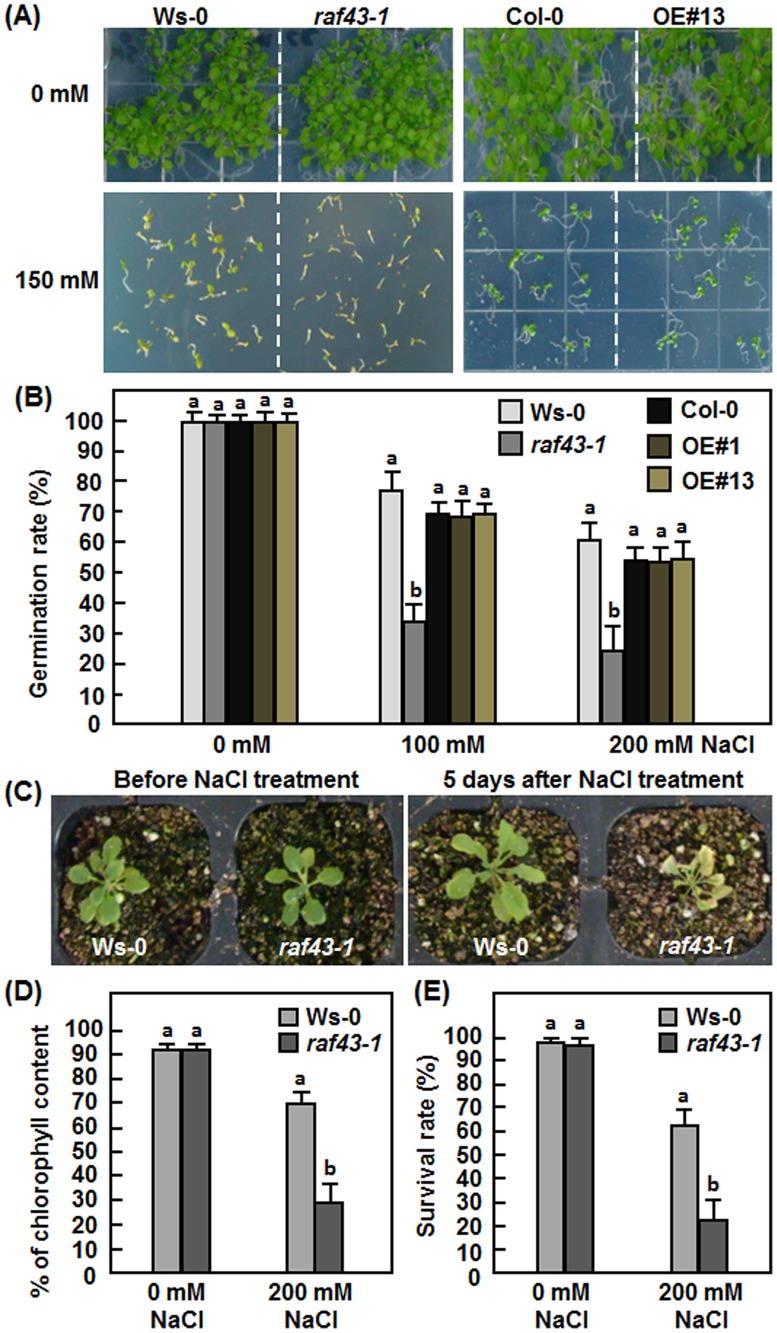
*Raf43* is required for salt stress tolerance. (A) and (B) Seed germination on 1/2 MS supplemented with different concentrations of NaCl. (A) Seed germination and seedling growth on 1/2 MS supplemented with 0 and 150 mM of NaCl. Photos were taken at 10 days after germination. (B) Germination rates of seeds on 1/2 MS supplemented with 0, 100 and 200 mM of NaCl. Seed germination was recorded when the radicles emerged completely from seed coat. (C)-(E) Phenotype, chlorophyll content and survival rates of soil-grown plants after NaCl treatment. (C) Phenotype of the wild type Ws-0 and mutant *raf43-1* plants under drought stress condition. Three-week-old soil-grown plants were drenched with 0 or 200 mM NaCl and allowed to grow for 10 days. Left photo shows the growth status of the plants before NaCl treatment while right photo shows phenotype of the plants at 5 days after NaCl treatment. (D) Percentages of chlorophyll content in leaf tissues of wild type Ws-0 and mutant *raf43-1* plants grown under normal and NaCl stress condition at 5 days after treatment. (E) Survival rates of wild type Ws-0 and mutant *raf43-1* plants grown under normal and drought stress condition at 10 days after treatment. Experiments were repeated three times and data presented in (B), (D) and (E) are the means ± standard errors from three independent experiments. Different letters above the columns indicate significant difference at *p* = 0.05 between Ws-0 and *raf43-1* plants and between Col-0 and *Raf43*-overexpressing plants.

Possible function of *Raf43* in salt stress tolerance was also evaluated by comparing the seed germination and root growth phenotype of the *raf43-1* mutant line and the Raf43-OE#1 and Raf43-OE#13 lines with their corresponding wild type Ws-0 and Col-0 after treatment with NaCl. Germination rates of seeds from the *raf43-1* mutant line, the Raf43-OE#1 and Raf43-OE#13 lines and their corresponding wild type Ws-0 and Col-0 was similar on NaCl-free 1/2MS medium but the germination rates of the *raf43-1*, Raf43-OE#1, Raf43-OE#13 and wild type seeds significantly decreased on 1/2MS medium supplemented with 100 mM or 200 mM NaCl as compared with those on mannitol-free medium (Fig 4A and 4B). In contrast, germination rates of the *raf43-1* mutant seeds markedly decreased on 1/2MS medium supplemented with 100 mM or 200 mM NaCl, resulting in 60% of reduction as compared with the Ws-0 seeds (Fig 4A). No significant difference in germination rates of seeds from the Raf43-OE#1 and Raf43-OE#13 lines and wild type Col-0 was observed (Fig 4A and 4B). We further compared the phenotype of three-week-old soil-grown *raf43-1* mutant and wild type Ws-0 plants in response to salt stress applied by drenching 200 mM NaCl. Before NaCl treatment, the growth status of the *raf43-1* and wild type Ws-0 plants was similar (Fig 4C, left); however, most of the leaves of the *raf43-1* plants showed yellowish necrotic symptoms, whereas only some leaves of the wild type Ws-0 plants were slightly yellowish at 5 days after NaCl treatment (Fig 4C, right). NaCl-induced damage in leaves of the *raf43-1* and wild type Ws-0 plants resulted in significant reduction of the chlorophyll contents as compared with those in untreated control plants (Fig 4D). The reduction of chlorophyll contents in the NaCl-treated leaves of the *raf43-1* plants was much higher than that in the wild type Ws-0 plants (Fig 4D). Chlorophyll content in leaves of NaCl-treated *raf43-1* plants decreased to 32% of that in leaves without NaCl treatment, whereas chlorophyll content in NaCl-treated leaves of the wild type Ws-0 was about 76% of that in leaves without NaCl treatment (Fig 4D). At 10 days after NaCl treatment, the survival rate of the *raf43-1* plants (21%) was markedly decreased as compared with that of the wild type Ws-0 plants (61%), showing a reduction of 66% (Fig 4E). No difference was observed in survival rates of the *raf43-1* and Ws-0 plants (>95%) without NaCl treatment during the 7-day experimental period (Fig 4E). These observations indicate that knockout of *Raf43* attenuated the salt stress tolerance whereas overexpression of *Raf43* had no effect, suggesting that *Raf43* is also required for salt stress tolerance in Arabidopsis.

### *Raf43* is required for oxidative stress tolerance

**Fig 5:**
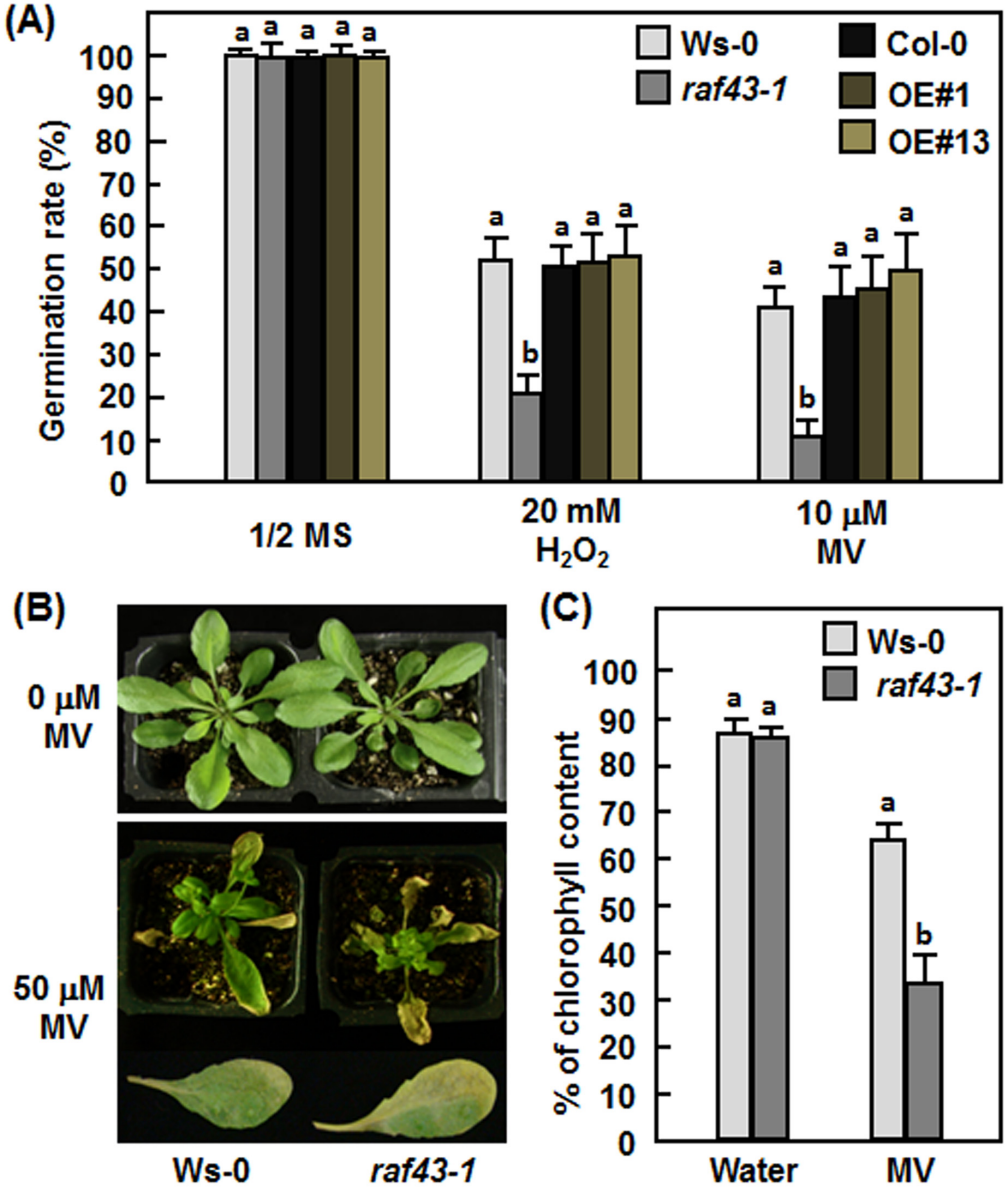
*Raf43* is required for oxidative stress tolerance. (A) Germination rates of seeds on 1/2 MS supplemented with 20 mM H_2_O_2_ or 10 μM methyl viologen (MV). Seed germination was recorded when the radicles emerged completely from seed coat. (B) and (C) Phenotype and chlorophyll contents in leaf tissues of the wild type Ws-0 and mutant *raf43-1* plants after treatment with 50 μM MV. Representative leaves showing typical yellowish and necrotic symptom from MV-treated Ws-0 and *raf43-1* plants were also shown. Three-week-old soil-grown plants were treated by foliar spraying with 50 μM MV or water as a control and allowed to grow for 7 days. Photos were taken at 5 days after treatment and leaf samples were collected for analyzing chlorophyll contents. Experiments were repeated three times and data presented in (A) and (C) are the means ± standard errors from three independent experiments. Different letters above the columns indicate significant difference at *p* = 0.05 between Ws-0 and *raf43-1* plants and between Col-0 and *Raf43*-overexpressing plants.

Because expression of *Raf43* was induced by oxidative stress (Fig 1), we thus examined whether mutation or overexpression of *Raf43* would affect tolerance to oxidative stress during seed germination or in mature Arabidopsis leaves. In 1/2MS medium without supplementation of MV or H_2_O_2_, seeds of the *raf43-1* mutant and the Raf43-OE#1 and Raf43-OE#13 lines showed similar germination rates to those of the corresponding wild type Ws-0 and Col-0 seeds (Fig 5A). In 1/2MS medium supplemented with 20 mM H_2_O_2_ or 10 μM MV, germination rates of seeds from the *raf43-1* mutant line, the Raf43-OE#1 and Raf43-OE#13 lines and the wild types Ws-0 and Col-0 lines were decreased significantly, but seeds of the *raf43-1* mutant line showed a lower germination rate than the wild type Ws-0 seeds (Fig 5A). At 6 days, only 20% and 10% of the *raf43-1* mutant seeds had germinated in the presence of 20 mM H_2_O_2_ or 10 mM MV, respectively, while 40–50% of wild type Ws-0 seeds had germinated (Fig 5A). By contrast, germination rates for seeds of the Raf43-OE#1 and Raf43-OE#13 lines in the presence of 20 mM H_2_O_2_ or 10 μM MV were comparable to those of the wild type Col-0 seeds (Fig 5A). We further assessed the oxidative stress tolerance in mature leaves of the *raf43-1* and wild type Ws-0 plants to MV. Without treatment with MV, both the *raf43-1* and wild type Ws-0 plants grew well; however, exogenous application of 50 μM MV by foliar spraying caused significant necrosis or bleaching, indicative of oxidative damage, on leaves of soil-grown four-week-old *raf43-1* and Ws-0 plants (Fig 5B). Oxidative damage caused by exogenously applied MV on leaves of the *raf43-1* plants was much more pronounced than on leaves of the wild type Ws-0 plants (Fig 5B). MV-induced oxidative damage in leaves of the *raf43-1* and wild type Ws-0 plants resulted in significant reduction of the chlorophyll contents as compared with those in untreated control plants (Fig 5C). The reduction of chlorophyll contents in the MV-treated leaves of the *raf43-1* plants was much evident than that in the wild type Ws-0 plants (Fig 5C). Chlorophyll content in MV-treated leaves of the *raf43-1* plants decreased to 39% of that in leaves without MV treatment, whereas chlorophyll content in MV-treated leaves of the wild type Ws-0 was about 71% of that in leaves without MV treatment (Fig 5C). These results indicate that knockout of *Raf43* could weaken oxidative stress tolerance, leading to an increased level of cellular damage under oxidative stress conditions but overexpression of *Raf43* did not affect the oxidative stress tolerance in Arabidopsis.

### *Raf43* is involved in ABA response

**Fig 6:**
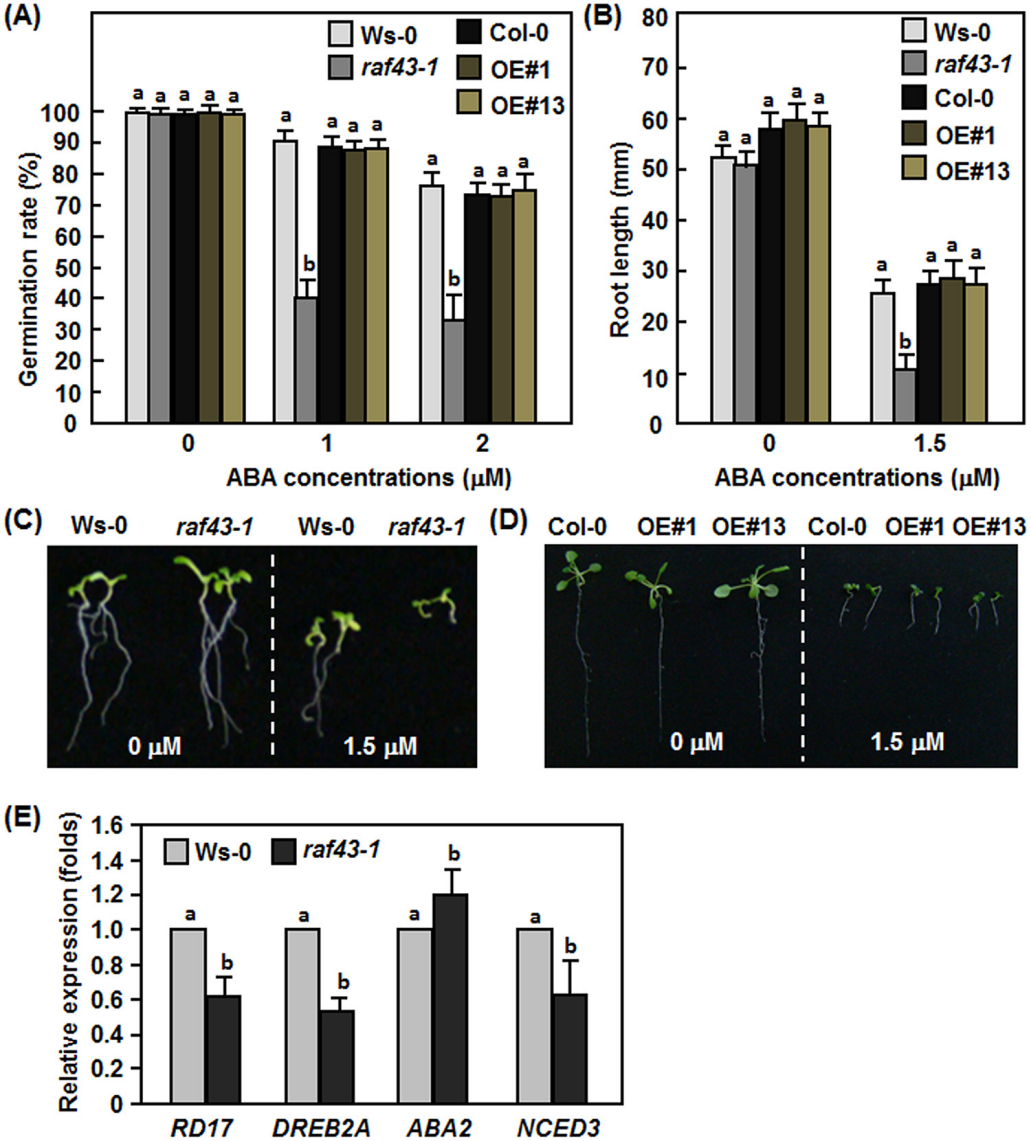
*Raf43* is involved in ABA response. (A) Seed germination rates on 1/2 MS supplemented with 0, 1 and 2 μM of ABA. Seed germination was recorded when the radicles emerged completely from seed coat. (B) Root length of seedlings grown on 1/2 MS supplemented with 0 and 1.5 μM of ABA. (C) and (D) Root phenotype of seedlings grown on 1/2 MS supplemented with 0 and 1.5 μM of ABA. (E) Expression of stress-responsive and ABA biosynthesis-related genes in WS-0 and *raf43-1* plants. Leaf samples were collected form four-week-old soil-grown plants for analyzing gene expression through qRT-PCR. Expression data were normalized by the transcript level of *Ubiquitin*. The expression levels of *Raf43* in wild type Ws-0 plants was set as 1 and the levels in *raf43* mutant plants are shown as folds of the levels in wild type plants. Experiments were repeated three times and data presented in (A), (B) and (E) are the means ± standard errors from three independent experiments. Different letters above the columns indicate significant difference at *p* = 0.05 between Ws-0 and *raf43-1* plants and between Col-0 and *Raf43*-overexpressing plants.

Considering that *Raf43* is required for multiple abiotic stress tolerance as mentioned above and that ABA is an important stress hormone involved in regulating abiotic stress response [49], we therefore examined the possible involvement of *Raf43* in ABA response. For this purpose, seed germination and root growth of the *raf43-1* mutant line and the Raf43-OE#1 and Raf43-OE#13 lines in response to exogenous ABA were assayed and compared with their corresponding wild types Ws-0 and Col-0. Germination rates of seeds from the *raf43-1* line, the Raf43-OE#1 and Raf43-OE#13 lines and their corresponding wild type Ws-0 and Col-0 were similar when germinated on ABA-free 1/2MS medium but the germination rates decreased significantly on 1/2MS medium supplemented with 1 μM or 2 μM ABA as compared with those on ABA-free medium (Fig 6A). However, germination rates of the *raf43-1* mutant seeds markedly decreased on 1/2MS medium supplemented with 1 μM or 2 μM ABA, resulting in 55–60% of reduction as compared with the Ws-0 seeds (Fig 6A). No significant difference in germination rates of seeds from the Raf43-OE#1 and Raf43-OE#13 lines and wild type Col-0 was observed in the presence of ABA (Fig 6A). After germination, root length of the *raf43-1* seedlings, the Raf43-OE#1 and Raf43-OE#13 seedlings and their corresponding wild type Ws-0 and Col-0 seedling was similar when grown on ABA-free 1/2MS medium but the root growth was significantly inhibited when grown on 1/2MS medium supplemented with 1.5 μM ABA as compared with those on ABA-free medium (Fig 6B–6D). Root growth of the *raf43-1* seedlings was significantly inhibited on 1/2MS medium supplemented with 1.5 μM ABA, leading to a 56% of reduction as compared with the Ws-0 seedlings (Fig 6B and 6C). No significant difference in root length of the Raf43-OE#1 and Raf43-OE#13 seedlings and the wild type Col-0 seedlings was observed (Fig 6B and 6D). These observations indicate that knockout of *Raf43* resulted in increased ABA sensitivity but overexpression of *Raf43* had no effect on ABA response in Arabidopsis.

To gain information on the possible mechanism of action of *Raf43* in abiotic stress response, we analyzed through qRT-PCR and compared the expression of four selected stress-responsive and ABA biosynthesis-related genes such as *NCED3* [50], *ABA2* [51, 52], *RD17* (*COR47*) [53], and *DREB2A* [54] in *raf43-1* and Ws-0 plants grown under normal condition. As shown in Fig 6E, the expression levels of the stress-responsive *RD17* and *DREB2A* in *raf43-1* plants were significantly reduced, leading to 50% and 40% of reduction, respectively, as compared with those in Ws-0 plants (Fig 6E). The ABA biosynthetic genes *NCED3* and *ABA2* showed different expression patterns in the *raf43-1* plants. The expression level of NCED3 in the *raf43-1* plants reduced markedly, leading to a 39% of reduction whereas the expression level of *ABA2* showed a slight increase (19%), as compared with those in Ws-0 plants (Fig 6E). These results indicate that knockout of *Raf43* resulted in altered expression of stress-responsive and ABA biosynthesis-related genes.

### *Raf43* is not required for resistance against *B*. *cinerea* and *P*. *syringae* pv. *tomato* DC3000

**Fig 7:**
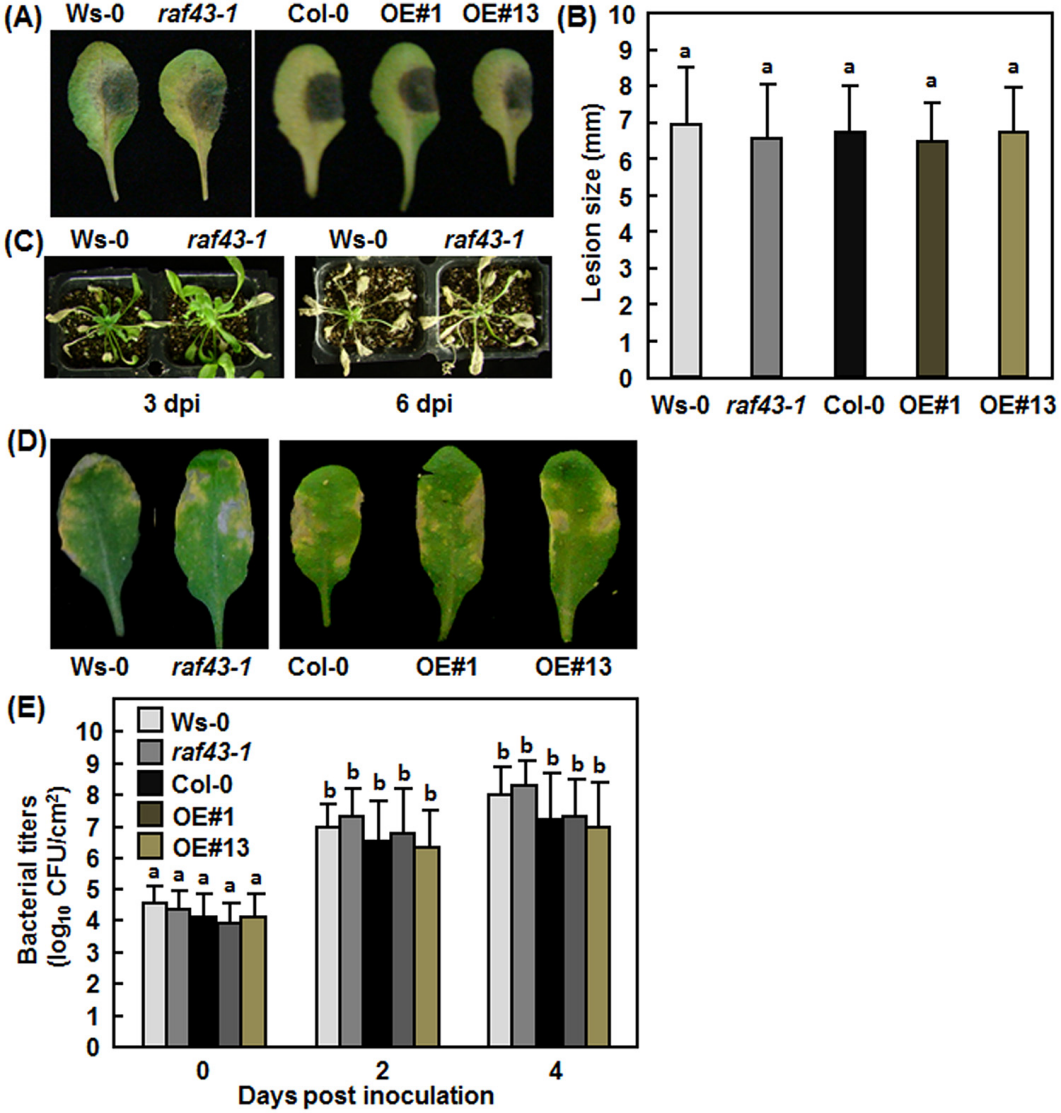
*Raf43* is not required for resistance to *B*. *cinerea* and *P*. *syringae* pv. *tomato* DC3000. (A)-(C) Phenotype of disease caused by *B*. *cinerea*. (A) Disease phenotype on leaves of different genotype plants in detached leaf inoculation assays. (B) Lesion sizes on leaves from (A). Leaves were detached from four-week-old plants and inoculated by dropping 5 μL of *B*. *cinerea* spore suspension (1×10^5^ spores/mL). Photos were taken and lesion sizes were recorded at 5 days after inoculation. (C) Disease phenotype on wild type Ws-0 and mutant *raf43* plants. Four-week-old plants were inoculated by foliar spraying with *B*. *cinerea* spore suspension (1×10^5^ spores/mL) and photos were taken at 3 and 6 days after inoculation. dpi, days post inoculation. (D) and (E) Phenotype of disease caused by *P*. *syringae* pv. *tomato* DC3000. (A) Representative disease symptom on leaves of different genotype plants. (B) Bacterial titers in inoculated leaves. Four-week-old plants were inoculated by infiltration with *P*. *syringae* pv. *tomato* DC3000 (OD_600_ = 0.002). Photos were taken 4 days after inoculation. Leaf samples were collected at 0, 2, and 4 days after inoculation and bacterial growth was measured. Experiments were repeated three times and the data presented in (B) and (E) are the means ± standard errors from three independent experiments. Different letters above the columns indicate significant difference at *p* = 0.05 between Ws-0 and *raf43-1* plants and between Col-0 and *Raf43*-overexpressing plants.

The induction of *Raf43* by pathogen infection and elicitor treatment (Fig 1) led us to examine whether *Raf43* plays a role in resistance against different pathogens. Detached leaf inoculation and whole plant inoculation assays were performed to compare the disease phenotype of the *raf43-1* mutant plants and the Raf43-OE#1 and Raf43-OE#13 plants with their corresponding wild type plants after inoculation with *B*. *cinerea*. In the detached leaf inoculation assays, no significant difference in size of necrotic lesions caused by *B*. *cinerea* was observed between Ws-0 and *raf43-1* plants and between Col-0 and Raf43-OE#1 and Raf43-OE#13 plants (Fig 7A and 7B). Similar results were also obtained from the whole plant inoculation assays. Typical yellowish necrotic lesions were observed on leaves of wild type Ws-0 and mutant *raf43-1* plants at 3 days and these diseased plants started to die at 6 days after inoculation by foliar spraying of *B*. *cinerea* spores suspension (Fig 7C). On the other hand, we also explored the possible function of *Raf43* in resistance against a bacterial pathogen by comparing disease phenotypes of the *raf43-1* mutant plants and the Raf43-OE#1 and Raf43-OE#13 plants with their corresponding wild types Ws-0 and Col-0 plants after inoculation with a normally virulent strain of *P*. *syringae* pv. *tomato* DC3000. In these assays, typical chlorotic lesions were observed in inoculated leaves of the Ws-0 and *raf43-1* plants and the Col-0 and Raf43-E#1/Raf43-OE#13 plants at 4 days after inoculation (Fig 7D). Measurement of bacterial titers in inoculated leaves showed no significant difference in bacterial proliferation between the Ws-0 and *raf43-1* plants and the Col-0 and Raf43-OE#1/Raf43-OE#13 plants at 2 and 4 days after inoculation (Fig 7E). These results indicate that knockout or overexpression of *Raf43* did not affect the response of Arabidopsis plants to infection of *B*. *cinerea* and *P*. *syringae* pv. *tomato* DC3000, implying that Raf43 is not required for resistance to these two pathogens.

### Native promoter-driven expression of *Raf43* restores the stress tolerance in *raf43* plants

**Fig 8:**
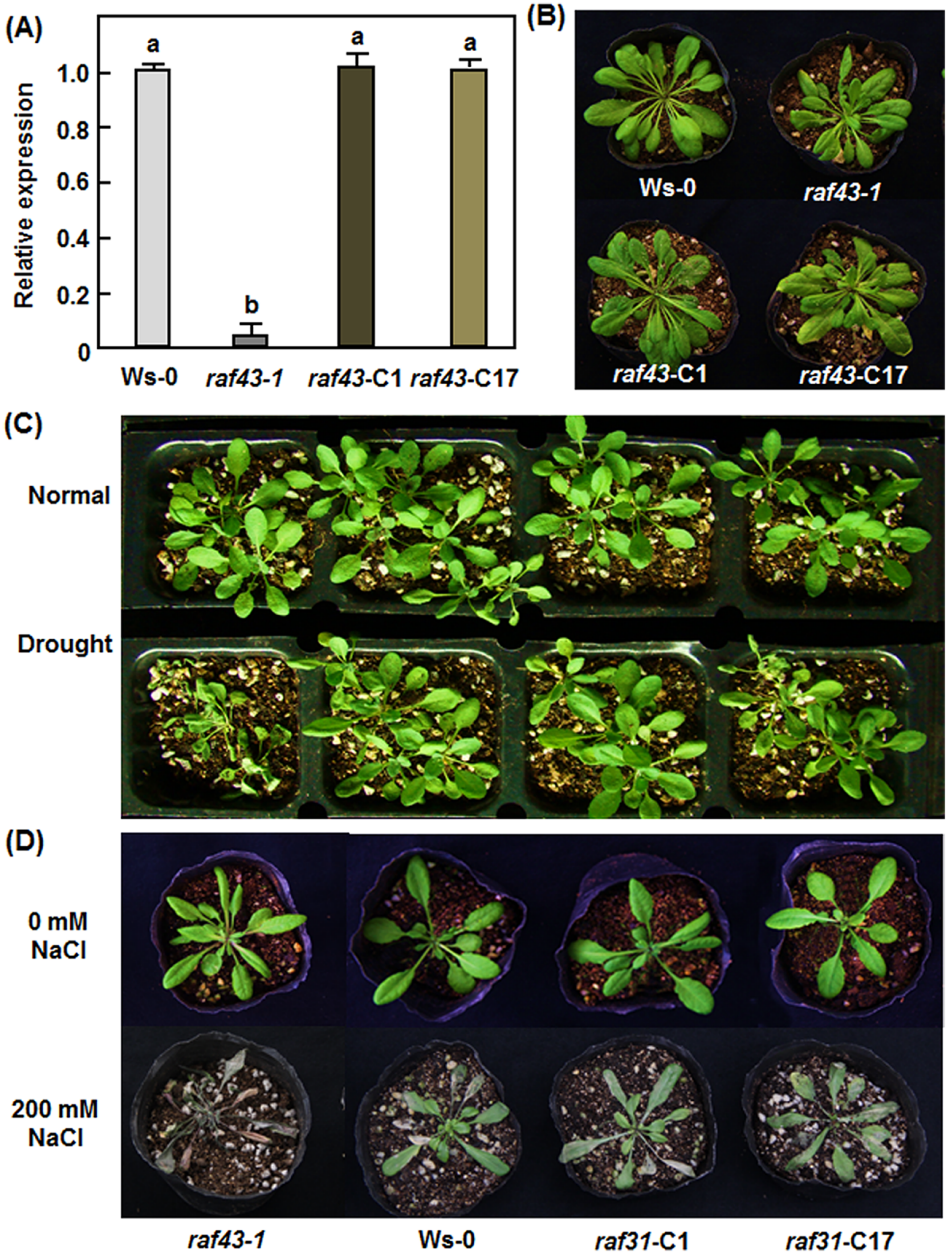
Native promoter-driven expression of *Raf43* complemented the phenotype of *raf43-1* plants under stress conditions. (A) Levels of the *Raf43* transcripts in *Raf43*-complementation lines Raf43-C1 and Raf43-C17 plants. The transcript levels of *Raf43* in four-week-old plants grown under normal conditions were analyzed by qRT-PCR using *Raf43*-specific primers and data were normalized by the transcript level of *Ubiquitin*. The transcript level of *Raf43* in Ws-0 plants was set as 1 and the levels in *raf43-1*, Raf43-C1 and Raf43-C17 plants are shown as folds of the levels in Ws-0 plants. Data represented are the means ± standard errors from three independent experiments and different letters above the columns indicate significant difference at *p* = 0.05. (B) Morphological and growth phenotypes of four-week-old Ws-0, *raf43-1*, Raf43-C1 and Raf43-C17 plants. (C) Phenotype of the Ws-0, *raf43-1*, Raf43-C1 and Raf43-C17 plants under drought stress condition. Three-week-old soil-grown plants were withheld from watering for drought stress treatment and allowed to grow for 7 days. Photo shows the phenotype of the plants at 7 days after drought stress treatment. (D) Phenotype of the Ws-0, *raf43-1*, Raf43-C1 and Raf43-C17 plants under salt stress condition. Three-week-old soil-grown plants were drenched with 200 mM NaCl and allowed to grow for 7 days. The photo shows phenotype of the plants at 7 days after NaCl treatment. Repeated experiments for (C) and (D) showed similar results.

Because only one T-DNA insertion line was used for analysis of the *Raf43* function, genetic complementation was thus carried out to determine whether the decreased abiotic stress tolerance that we observed in *raf43* plants is indeed the result of the mutation within the *Raf43* gene. A fusion of 1295 bp native promoter region and the *Raf43* ORF was introduced into *raf43-1* mutant background. Homozygous T3 lines with a single copy of the transgene were screened based on a 3:1 Hgr-resistant/Hgr-sensitive segregation ratio and two independent complementation lines, named *Raf43*-C1 and *Raf43*-C17, were chosen for further studies. qRT-PCR analysis showed that the transcript levels of *Raf43* in Raf43-C1 and Raf43-C17 plants were comparable to that in Ws-0 plants but significantly higher than that in *raf43-1* plants (Fig 8A). The growth and morphology of the Raf43-C1 and Raf43-C17 plants were undistinguishable from the Ws-0 and *raf43-1* plants (Fig 8B). In drought and salt stress assays, the Raf43-C1 and Raf43-C17 plants displayed similar phenotypes while the *raf43-1* plants showed significant stress damages, as compared with the Ws-0 plants (Fig 8C and 8D). These data indicate that the native promoter-driven expression of *Raf43* fully complemented the phenotypes observed in the *raf43-1* plants, demonstrating that the decreased abiotic stress tolerance in *raf43* plants is indeed caused by the mutation within the *Raf43* gene.

## Discussion

The plant MAPKKKs have great sequence diversity and are categorized either as MEKKs, Raf and ZIK subfamilies or as three groups (Groups A, B and C) [8, 10]. However, only a few of Raf-like MAPKKKs in plants have been studied and most of the characterized Raf-like MAPKKKs belong to the Group B of Raf-like MAPKKKs, including Arabidopsis CTR1, EDR1, SIS8/AT6 and MAP3Kδ4 and rice ERD1 and DSM1 [32, 33, 39–42, 44, 55]. To date, the sole Group C Raf-like MAPKKK that has been functionally characterized in plants is the rice ILA1 [43]. In the present study, we identified an Arabidopsis stress-responsive Group C Raf-like MAPKKK gene, *Raf43*, which can be induced by multiple abiotic and biotic stresses. Functional analyses using a T-DNA insertion mutant *raf43-1* demonstrated that *Raf43* is required for tolerance to multiple abiotic stresses including drought, osmotic, salt and oxidative stresses. Our findings provide new insights into the biological roles of the functionally uncharacterized Group C Raf-like MAPKKKs in plants.

Our interesting on *Raf43* came from bioinformatics analyses of publicly available microarray data to identify putative stress-responsive MAPKKK genes. Expression profiling analyses using publicly available microarray data and our qRT-PCR data clearly demonstrated that *Raf43* is responsive to multiple abiotic stresses including drought, oxidative, osmotic and salt stress (Fig 1). Phenotype analyses of the *raf43-1* mutant and two *Raf43*-overexpressing lines revealed that *Raf43* is necessary but not sufficient for tolerance to drought, osmotic, oxidative and salt stresses and thus acts as a positive regulator of multiple abiotic stress responses. This is different from the function of SIS8/AT6, which negatively regulates salt tolerance in Arabidopsis [39]. Several lines of evidence presented in this study support the requirement of *Raf43* for tolerance to abiotic stress response. Firstly, seed germination and seedling root growth of *raf43-1* line was significantly inhibited on medium supplemented with mannitol, NaCl, H_2_O_2_ or MV (Figs 3, 4 and 5). Secondly, reduced tolerance to drought, salt and oxidative stress was observed in the soil-grown mature *raf43-1* plants upon withholding of water, drenching of NaCl solution or foliar spraying of MV (Figs 3, 4 and 5). This is in agreement with the function of rice DSM1, which was found to positively regulate drought and oxidative stress tolerance [42]. The native promoter-driven expression of *Raf43* fully complemented the phenotypes of the *raf43-1* plants under abiotic stress conditions, demonstrating that the decreased abiotic stress tolerance in *raf43-1* plants is indeed caused by the mutation within the *Raf43* gene (Fig 8). Thirdly, the expression of *RD17* and *DREB2A*, two stress-responsive genes [53, 54], was downregulated in *raf43-1* plants (Fig 6E), indicating that *Raf43* may be involved in stress-responsive signaling pathway. Fourthly, mutation in *Raf43* resulted in increased ABA sensitivity as seed germination and seedling root growth of *raf43-1* line was inhibited markedly in the presence of exogenous ABA (Fig 6). It is well known that, as a stress hormone, ABA plays a key role in the regulation of many physiological processes in response to abiotic stress response [49, 56]. The altered expression of *NCED3* and *ABA2*, two stress-responsive genes [50–52], in *raf43-1* plants may indicate the involvement of *Raf43* in ABA response as well as in ABA-mediated signaling pathway. This differs from the function of SS8/AT6 that negatively regulates salt stress tolerance in an ABA-independent manner [39]. In addition, our functional analyses in this study also demonstrate that *Raf43* has pleiotropic effects on tolerance to multiple abiotic stresses. This is similar to SIS7/AT6, which was shown to play roles in salt and high sugar response [39, 40], and to rice DSM1, which is required for both drought and oxidative stress [42].

Both microarray data- and qRT-PCR-based expression profiling analyses indicated that the expression of *Raf43* was significantly induced by infection of some fungal and bacterial pathogens and treatment with elicitors including some well-known pathogen-associated molecular patterns and defense signaling hormone SA (Fig 1) [57], implying a role for *Raf43* in disease resistance. Surprisingly, neither the *raf43-1* plants nor the *Raf43*-overexpressing plants exhibited any difference in disease phenotypes to the wild type plants after inoculation with necrotrophic fungal pathogen *B*. *cinerea* and hemibiotrophic bacterial pathogen *P*. *syringae* pv. *tomato* DC3000 (Fig 7). It is thus unlikely that *Raf43* plays a role in resistance against these two pathogens. However, the involvement of *Raf43* in biotic stress response cannot be ruled out at present and needs to be investigated further using more pathogens with different infection styles. Furthermore, the significant induction of *Raf43* by some of pathogen-associated molecular patterns such as FLG22 and EF-Tu may imply that *Raf43* has a function in regulating innate immunity in Arabidopsis.

It was previously reported that overexpression of tobacco *NPK1* and Arabidopsis MAP3Kδ4 in stable transgenic plants and transient expression of tomato *MAPKKKε* and *N*. *benthamiana NbMAPKKKα*, *NbMAPKKKβ* and *NbMAPKKKγ* in leaves of *N*. *benthamiana* resulted in increased tolerance to abiotic stresses and accelerated programmed cell death, respectively [28, 30, 55, 58, 59]. Similarly, overexpression of *DSM1*, encoding for a Group B Raf-like MAPKKK, in transgenic rice increased the tolerance to drought stress at the seedling stage [42]. However, we did not observe any alteration in abiotic and biotic stress response in the two *Raf43*-overexpressing lines. The reason for this phenomenon is unclear. The possibility that sequence errors of the transgene were introduced during the cloning and vector construction processes and thus resulted in misexpression of *Raf43* in *Raf43*-overexpressing lines can be ruled out as no any error in the transgene was identified in these overexpression lines by re-sequencing. The most possibility to explain the phenomenon that overexpression of *Raf43* did not result in any alteration in abiotic stress response should be associated with the biochemical nature of the Raf43 protein itself. Some of Raf-like MAPKKKs such as Arabidopsis CTR1 and rice ILA1 have been reported to possess kinase activities and have autophosphorylation activity *in vitro* [43, 60, 61]. It was also found recently that phosphorylation of Arabidopsis MEKK1 via Ca^2+^ signaling is critical to its function in cold stress response [62]). Furthermore, the kinase catalytic domains in Raf-like MAPKKKs including Arabidopsis CTR1 and EDR1 and rice DSM1 were shown to have kinase activity [42, 60, 61]. Raf43 contains a typical kinase catalytic domain at C-terminal (Fig 2B). It is thus likely that the activity of Raf43 as a predicted MAPKKK needs to be activated via phosphorylation by itself or upstream signals and thus simply increases of the transcript or protein through the overexpression way cannot exert its biochemical function in the signaling events. Similar phenomenon was also observed for some MAPKKs. For example, overexpression of *MKK3* in Arabidopsis or transient expression of tomato *SlMKK2* and *SlMMK4* in *N*. *benthamiana* did not affect the disease phenotype of *P*. *syringae* pv. *tomato* DC3000 and the appearance of programmed cell death, respectively, whereas overexpression of constitutively active forms of these MAPKKs conferred clear phenotypes [63, 64]. Alternatively, the kinase activity of full-length Raf43 may be another factor that affects its kinase activity in the *Raf43*-overexpressing plants. It was found that the full-length Arabidopsis MEKK1 showed almost no activity but the kinase domain of MEKK1 had a strong ability to phosphorylate its substrate MKK2 [62]. Further studies on biochemical characterization of the kinase activity of Raf43 and phenotypical analyses of plants overexpressing the kinase domain of Raf43 will be helpful to address these questions.

As components acting at the top of MAPK cascades, MAPKKKs are generally believed to phosphorylate their downstream MAPKKs to relay the signal transduction events. In this regard, several members of the MEKK group have been shown to function as MAPKKKs upstream of MAPKKs in MAPK cascades. It has been demonstrated that the Arabidopsis MEKK1 and YODA and the tobacco NPK1, all belonging to the MEKK group of MAPKKKs, can form typical MAPK cascades, e.g. MEKK1–MKK4/MKK5–MPK3/MPK6 [65], YDA—MKK4/MKK5–MPK3/MPK6 [18, 66], and NPK1–NQK1–NRK1 [67]. However, traditional MAPK cascade has not been established yet for members of the Raf-like MAPKKKs. Instead, several studies have indicated that some of the identified Raf-like MAPKKKs do not function as MAPKKKs. For example, CTR1 was reported to inhibit MKK9–MPK3/MPK6 activation [36], whereas EDR1 was found to negatively regulate the MKK4/MKK5-MPK3/MPK6 cascade [38]. In our yeast two-hybrid-based pairwise interaction experiment, no significant interaction of Raf43 to any of 10 Arabidopsis MAPKKs was detected (data not shown). This is in agreement with the observation that the rice ILA1, a group C of Raf-like MAPKKK, did not interact with all rice MAPKKs [43]. Thus, it is likely that members of the Raf-like MAPKKKs may not follow the traditional MAPK cascades; instead, they exert their function by directly interacting with specific targets, which are not MAPKKs. This hypothesis is supported by the identification of the ILA1- and SIS8/AT6-interacting proteins. ILA1 was shown to interact with six closely related proteins with unknown function while SIS8/AT6 was found to interact with a UDP-glucosyltransferase UGT72E1 [40, 43]. Another, direct phosphorylation of a transcription factor by a MAPKKK has also been previously reported [68]. Therefore, it is possible that Raf43 may interact with downstream targets rather than MAPKKs to modulate the signaling events. Further screening and identification of Raf43-interacting proteins will provide new information on the mechanism for the function of Raf43 in abiotic stress response.
